# Recurrent Left Ureterosciatic Herniation With Obstructive Hydronephrosis: A Case Report

**DOI:** 10.1155/criu/1012541

**Published:** 2026-09-25

**Authors:** Venkatesh Perumalla, Thomas Neerhut, Eve Brown, John Blazak, Chandra Perumalla, Abigail Attwell-Heap

**Affiliations:** ^1^ Department of Urology, Sunshine Coast University Hospital, Birtinya, Australia, health.qld.gov.au; ^2^ Deakin University School of Medicine, Geelong, Australia; ^3^ Department of Medical Imaging, Sunshine Coast University Hospital, Birtinya, Australia, health.qld.gov.au

**Keywords:** case report, hydronephrosis, piriformis, ureteric hernia, ureterolysis

## Abstract

Uretero‐sciatic herniation is an exceptionally rare cause of ureteric obstruction, resulting from herniation of the ureter via the sciatic foramen. A 66‐year‐old woman presented with left flank pain and deteriorating renal function. Computed tomography intravenous pyelogram (CT IVP) demonstrated severe left hydronephrosis secondary to herniation of the ureter through the left sciatic foramen. The patient then underwent successful renal decompression with an endoscopic retrograde ureteric stent placed. Subsequent trial of stent removal failed, with left uretero‐sciatic herniation and renal obstruction recurring shortly after stent removal. Multidisciplinary review led to further evaluation for potential underlying pathology and subsequent definitive open ureterolysis, ureteric reduction and omental wrapping being performed. This case report highlights the pertinent clinical, radiological and management principles underlying such rare pathology. The possible association of piriformal atrophy as an underlying precipitant is discussed along with the key radiological findings of such rare pathology. Within the literature, evidence‐based definitive management options are limited and currently require multidisciplinary discussion with invasive surgical correction to be considered following failure of more minimally invasive endoscopic management. In this case, given the success of open ureterolysis and omental wrapping, it is hoped future cases may benefit from this knowledge and consider similar surgical approaches to achieve definitive management.

## 1. Case Presentation

### 1.1. Introduction

Pelvic floor hernias are rare, with sciatic hernias representing the least common subtype [1]. Ureteral involvement in sciatic herniation is exceedingly uncommon, with the majority of ureteral hernias occurring in the inguinal canal. Only a limited number of ureterosciatic hernia cases have been described in the literature. Here, we describe a rare case of recurrent left ureterosciatic herniation with urinary obstruction. The potential link with piriformal atrophy, relevant diagnostic clinical and radiological findings are discussed, as well as the current evidence regarding management of this rare pathology.

### 1.2. Case Presentation

**Figure 1:**
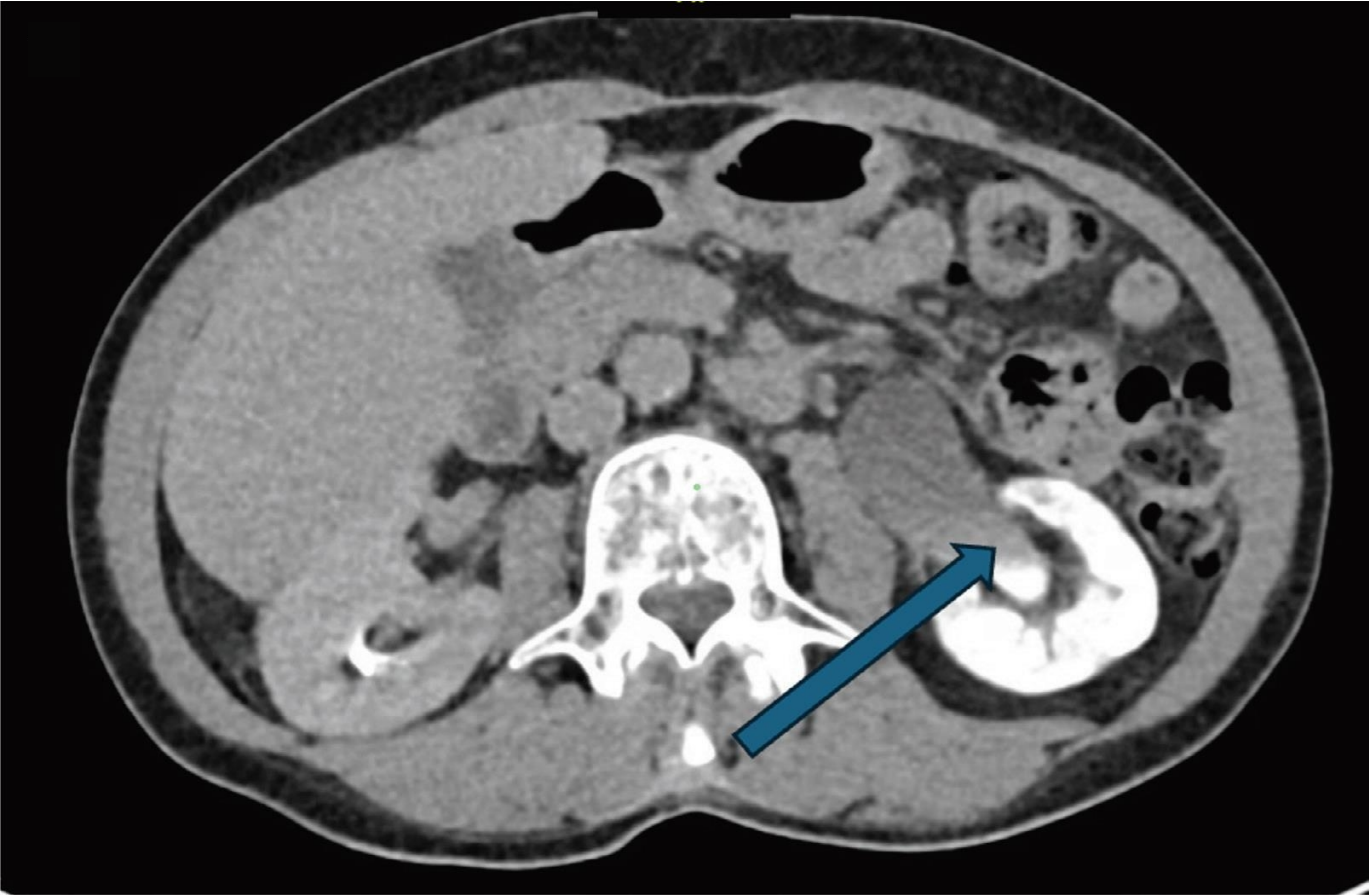
Postcontrast CT axial image demonstrating a delayed nephrogram involving the left kidney (blue arrow). A finding suggestive of high‐grade ureteric obstruction.

**Figure 2:**
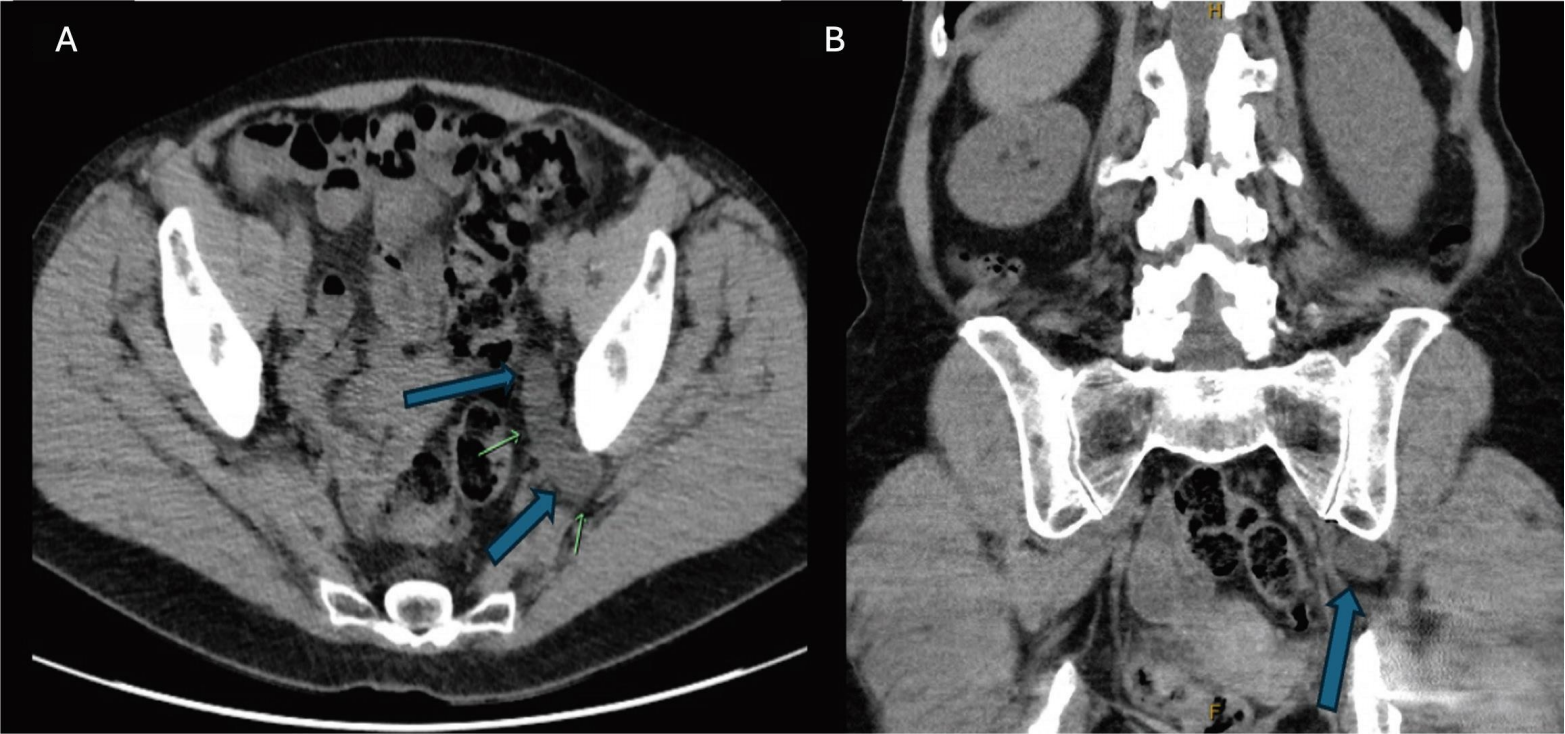
(A,B) Noncontrast (A) CT axial and (B) coronal images demonstrating the distal left ureter traced to the level of the left greater sciatic foramen through which it has herniated and abruptly tapers (blue arrows).

**Figure 3:**
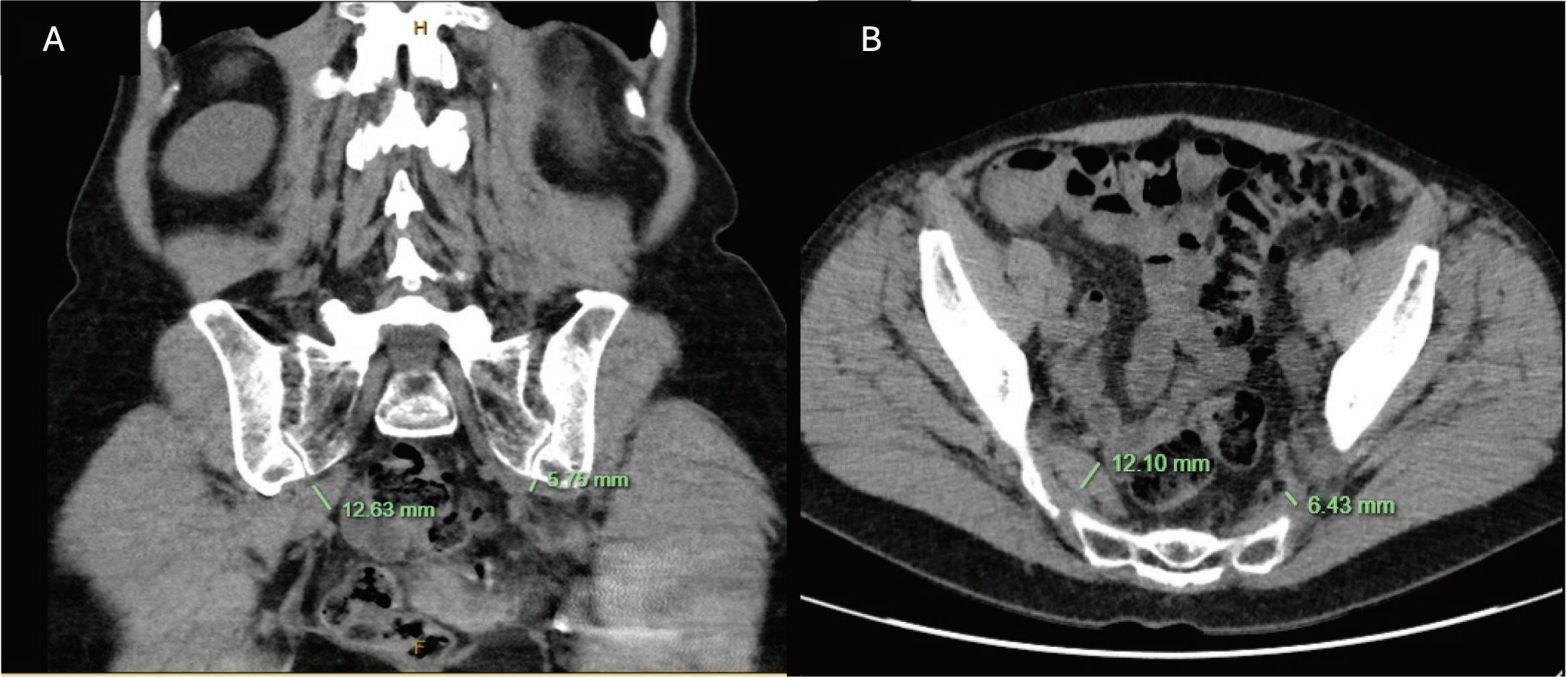
(A,B) Noncontrast CT (A) coronal and (B) axial images demonstrating significant atrophy of the left piriformis when compared with the right. Measurements were taken in both the coronal and axial plane utilising multiplanar reformats and Hounsfield attenuation values to ensure measurements were taken at identical anatomical levels bilaterally. The green markers indicate muscle bulk differential (12.63 vs. 5.75 mm in the coronal plane, 12.10 vs. 6.43 mm in the axial plane).

A 66‐year‐old patient presented to the emergency department with a 4‐week history of progressive left flank pain and declining renal function. The patient′s BMI was 23. Other than a recent left total hip replacement (THR) 4 months prior to presentation, the patient had no other significant past medical history. CT IVP demonstrated severe left hydronephrosis with delayed nephrogram (Figure 1). Despite lack of contrast opacification within distal the left ureter due to significant hydroureteronephrosis, the ureter could be traced distally adjacent to the left piriformis muscle and greater sciatic foramen where abrupt narrowing was noted. Findings were most consistent with ureterosciatic herniation (Figure 2A,B). Interestingly, significant left atrophy of the left piriformis was also noted on noncontrast imaging (Figure 3A,B).

### 1.3. Investigations and Initial Intervention

**Figure 4:**
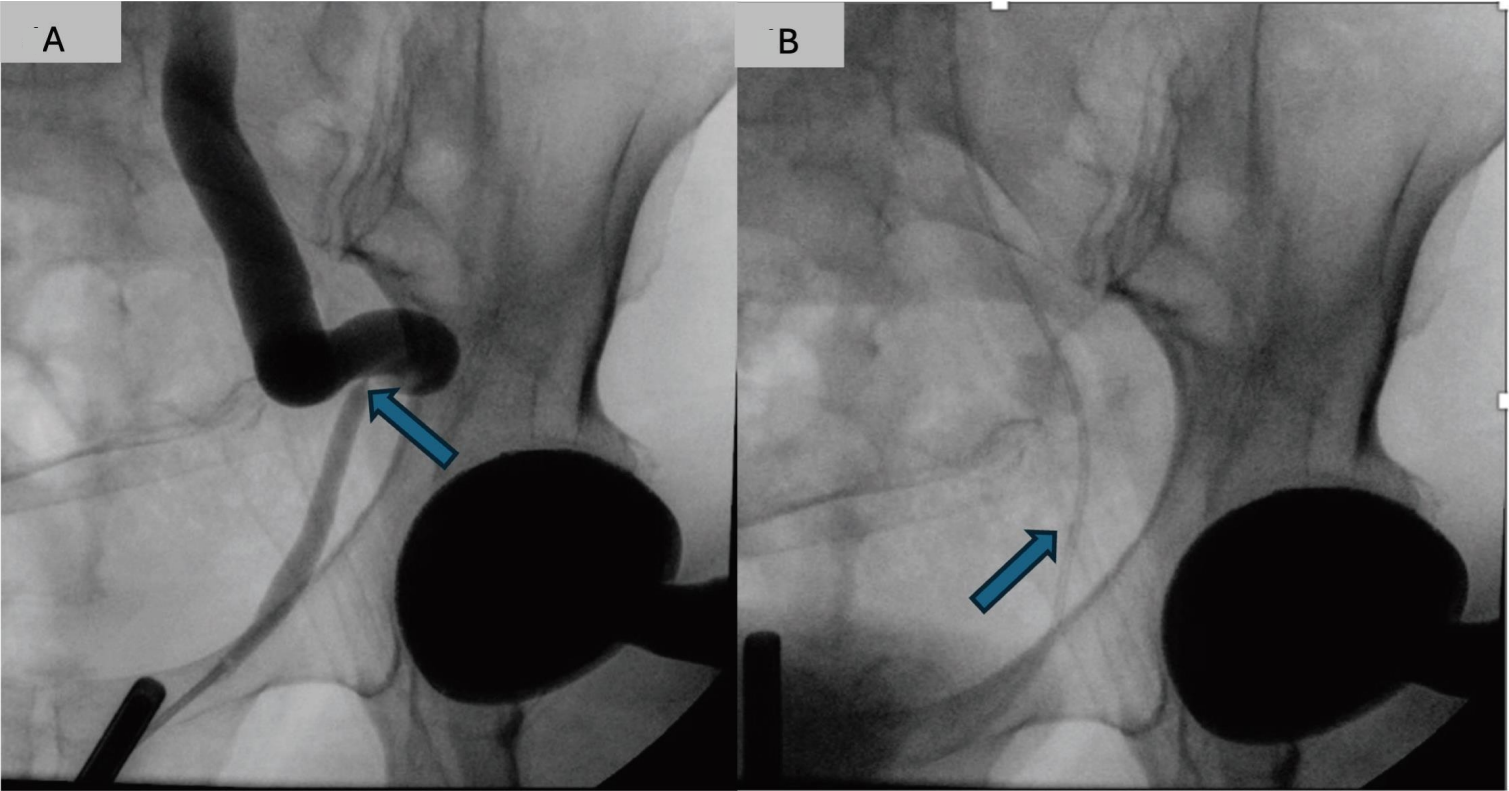
(A,B) (A) Intraoperative retrograde pyelogram with the blue arrow demonstrating the point of ureteric kinking with abrupt tapering at the approximate position of the left sciatic foramen. (B) Additional intraoperative fluoroscopic acquisition with the blue arrow pointing to the medialised and reduced left ureter following retrograde placement of the ureteric stent.

At the time of initial presentation, the patient′s serum creatinine was 100 umol/L and estimated glomerular filtration rate (eGFR) was 51 mL/min. Their baseline creatinine 4 months prior was 68 umol/L and eGFR 70 mL/min, thus confirming an acute kidney injury. Urine microscopy and culture was unremarkable. To achieve acute left renal decompression, the patient underwent cystoscopy and insertion of a left ureteric stent. Retrograde pyelograms were taken at the time again revealing a tortuous, laterally deviated distal ureter with an abrupt kink at the left sciatic foramen, the likely transition point (Figure 4A). Ureteral stent placement was straightforward and resulted in repositioning of the left ureter to its normal position (Figure 4B). The patient experienced improvement in symptoms and renal function following resolution of renal obstruction as demonstrated by a reduction in serum creatinine to 67 umol/L and an eGFR now having returned to the patient′s baseline of 70 mL/min. Six weeks later, given anatomical reduction of the ureteric hernia and complete resolution in symptoms, it was hoped permanent repositioning of the ureter may have been achieved, and thus the ureteric stent was removed under flexible cystoscopic guidance. Soon after, the patient again presented with left flank pain. Repeat CT imaging demonstrated recurrence of left hydroureteronephrosis necessitating repeat ureteric stent insertion.

### 1.4. Surgical Management, Outcome and Follow‐Up

In order to facilitate a more definitive plan, the case was subsequently reviewed at the urology/radiology multidisciplinary team meeting. A diagnostic ureteroscopy, endoscopic biopsy of the distal left ureter and exchange of the left ureteric stent was organised in order to exclude ureteric malignancy prior to considering more definitive surgical management options. Histology returned benign and MDT discussion recommended open ureterolysis, ureteric reduction and omental wrapping.

A Pfannenstiel incision was made and a pelvic preperitoneal space created. The prestented reduced pelvic left ureter was identified with a focus of reactive periureteric fibrotic tissue overlying an apparent fascial defect. Upon closer inspection the majority of periureteric fibrosis appeared to overlie the greater sciatic foraminal defect superior to the atrophied piriformis, likely explaining the recurrent urterosciatic herniation. No evidence of dense ureteric stricture disease or ischaemia was encountered. The pelvic ureter was then carefully mobilised with division of the surrounding fibrotic tissue utilising a combination of sharp and blunt dissection while preserving the apparent healthy ureteric adventitial tissue.

The adjacent omentum was then mobilised and utilised as a 360° tubular omental wrap around the pelvic ureteric segment, simultaneously covering the sciatic defect as an anatomic method of closure. Thus, the omental wrap was placed around the pelvic ureter, placed over the sciatic foraminal defect and secured with 2‐0 vicryl sutures to prevent hernia recurrence and facilitate healthy tissue healing. Ureteric reimplantation was not required given the reduced ureteric segment appeared healthy, now lay in a normal medialised anatomic position and was unlikely to herniate again given omental support. Favourable healing was anticipated. Peritoneal closure was then completed followed by fascial closure and endoscopic ureteric stent exchange to facilitate postoperative healing. The patient was subsequently discharged Day 1 postoperative, and no intraoperative or periprocedural complications were encountered.

Four weeks postoperatively, cystoscopic removal of the ureteric stent occurred, and follow‐up MAG3 renogram performed at 6 months revealed no functional obstruction with a normal split renal function of 48 (left): 52 (right). Additionally at 6 months, serum creatinine remains stable at 68umol/min and eGFR is close to the patient′s pre‐THR baseline measuring 69 mL/min. Importantly, the patient remains asymptomatic and without symptoms to suggest recurrence.

**Figure 5:**
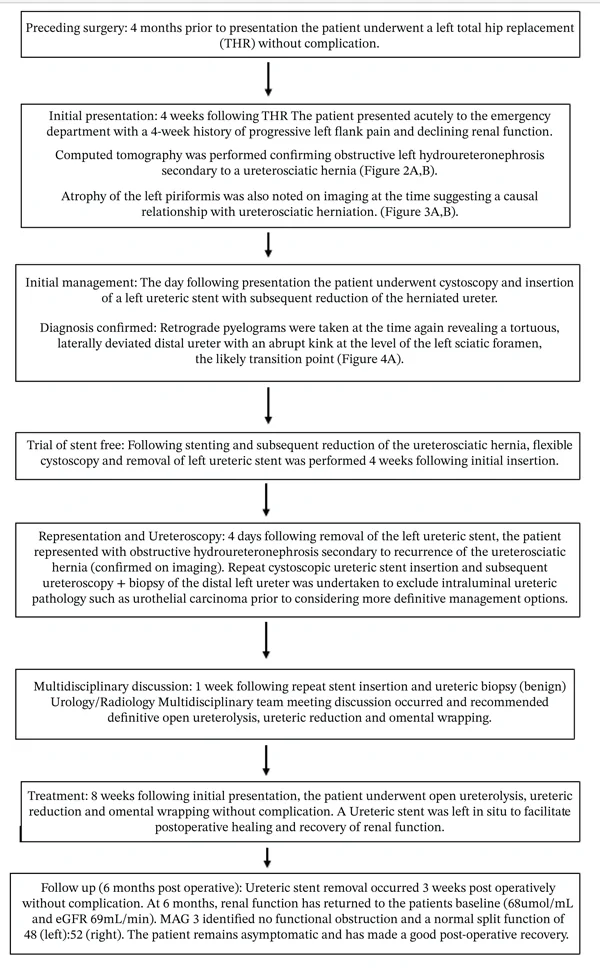
Case timeline.

A case timeline is provided (Figure 5).

## 2. Discussion

### 2.1. Epidemiology and Pathophysiology

Pelvic floor hernias are classified as obturator, perineal or sciatic, with the sciatic type being the rarest. Even more unusual is ureteral herniation through the sciatic foramen, which most commonly affects elderly women and predominantly involves the left ureter, the reason for which is currently unknown [2, 3].

Ureterosciatic herniation may arise due to atrophy of the piriformis muscle, hip joint disease or defects in the parietal pelvic fascia [4]. Atrophy of the piriformis muscle, is often seen in the context of a chronic increase in intra‐abdominal pressure such as during pregnancy, chronic constipation or trauma. The hernia may be classified anatomically according to the ureter′s relationship to the piriformis muscle as suprapiriformis, infrapiriformis or subspinous with the suprapiriformis variant being most common [5].

In our case, the patient had undergone a left THR just 4 months prior to presentation. The standard posterior THR approach involves tenotomizing the piriformis to gain access to the hip joint; this may result in significant atrophy of the muscle and thus predispose to ureterosciatic herniation. Several piriformis preserving posterior approaches have been described, such as the SPARE and STAR approach with improved reports of hip stability postoperatively [6]. Perhaps significantly, the patient presented was noted to have piriformal atrophy on initial imaging suggesting this may have contributed to the development of the ureterosciatic hernia (Figure 3A,B). Unfortunately, operative records from the patients THR and any preoperative imaging were not available for review. Additionally, the patient had no other risk factors for pelvic floor weakness. Thus, the relationship between THR, piriformal atrophy and urterosciatic herniation can merely be speculated. Further case series or retrospective observational analyses are required to establish a stronger link between these pathological entities.

### 2.2. Clinical Presentation

Clinical manifestations vary widely. Many patients are asymptomatic, and the diagnosis is often incidental. When symptomatic, flank pain, renal colic, urinary obstruction or neuropathic symptoms such as sciatica may occur [5].

### 2.3. Diagnosis

CT urography remains the diagnostic modality of choice, enabling visualization of the herniated ureter and associated hydronephrosis. Hydroureteronephrosis tapering at the point of obstruction within the sciatic foramen along with concurrent delayed nephrogram are typical findings. Other findings may be non‐specific, such as abnormal ureteric enhancement, which may be more suggestive of an inflammatory or malignant cause of obstruction. These require exclusion prior to consideration of definitive management.

Intraoperative fluoroscopy with iodinated contrast is a useful adjunct to further delineate the abnormal course of the herniated ureter in selected scenarios such as our case described.

### 2.4. Management Approaches

Management strategies are determined by symptom severity and recurrence. Ureteric stenting may offer temporary decompression, and several reports describe symptom resolution following stent insertion [2]. Some cases have instead opted for serial stent exchanges [7]. Definitive surgical correction has been described using open or laparoscopic approaches, including a combination of ureteric reimplantation, ureterolysis, and reduction of the herniated segment with reinforcement of the sciatic foramen [8]. More recently, ureterolysis combined with reduction of the herniated ureteric segment and repair of the sciatic defect either anatomically or through the use of an appropriate mesh has become the favoured definitive management option [8–11]. In our case, omental wrapping was utilised to anatomically support the reduced ureteric segment, a known successful method utilised following ureterolysis [12]. To date, no intraoperative or postoperative complications have been reported. Additionally, there are no cases of recurrences following this operative approach, suggesting this approach is an appropriate and safe definitive treatment option.

## 3. Conclusion

Ureterosciatic hernia is an exceedingly rare cause of recurrent ureteric obstruction. Cross‐sectional imaging is critical for diagnosis, and multidisciplinary management is recommended to guide appropriate intervention. Stenting may provide temporary relief, but recurrence after stent removal warrants consideration of definitive surgical repair which can be achieved with techniques such as ureterolysis. This case demonstrates the success of open ureterolysis and omental wrapping, the possible link with piriformal atrophy, the recurrent nature of the condition and the importance of MDT discussion to guide further diagnostic and therapeutic strategies.

## Author Contributions

Dr. Venkatesh Perumalla: conceptualisation, data collection and drafting and creation of final manuscript. Dr. Thomas Neerhut (co‐first author): conceptualisation, data collection and drafting and creation of final manuscript. Dr. Eve Brown: conceptualisation, initial draft, data collection and drafting and creation of final manuscript. Dr. John Blazak: drafting and creation of final manuscript. Dr. Chandra Perumalla: drafting and creation of final manuscript. Dr. Abigail Attwell‐Heap: drafting and creation of final manuscript.

## Funding

Open access publishing facilitated by Deakin University, as part of the Wiley‐Deakin University agreement via the Council of Australasian University Librarians.

## Disclosure

This manuscript has been read and approved by all the authors, that the requirements for authorship as stated earlier in this document have been met, and each author believes that the manuscript represents honest work.

## Ethics Statement

The protocol for this single case report was deemed low to negligible risk and consequently approved by the institution′s Human Research Ethics Committee conforming to the provisions of the Declaration of Helsinki.

## Consent

All informed consent was obtained from the subject discussed.

## Conflicts of Interest

The authors declare no conflicts of interest.

## Patient Perspective

Overall, I was happy with the treatment I received and am thankful the surgical team was able to solve a complex medical condition the way they did.

## Supporting information


**Supporting Information** Additional supporting information can be found online in the Supporting Information section.

## Data Availability

The data that support the findings of this study are available from the corresponding author upon reasonable request.
